# Clinicopathologic features of infection-related glomerulonephritis with IgA deposits: a French Nationwide study

**DOI:** 10.1186/s13000-020-00980-6

**Published:** 2020-05-27

**Authors:** Elodie Miquelestorena-Standley, Charlotte Jaulerry, Marie-Christine Machet, Nolwenn Rabot, Christelle Barbet, Aurélie Hummel, Alexandre Karras, Cyril Garrouste, Thomas Crepin, Didier Ducloux, Maud Cousin, Catherine Albert, Joseph Rivalan, Emilie Cornec-Le Gall, François Pourreau, Clément Deltombe, Dominique Nochy, Nora Szlavik, Sophie Felix, Anne Croué, David Buob, Nathalie Rioux-Leclerc, Laurent Doucet, Jean-Michel Goujon, Karine Renaudin, Emmanuelle Blanchard, Sébastien Eymieux, Marion Rabant, Jean-Michel Halimi

**Affiliations:** 1 Service d’anatomie et cytologie pathologiques, Hôpital Trousseau, CHRU Tours, Tours, France; 2 Université de Tours, PRES Centre-Val de Loire, Tours, France; 3 Service de néphrologie, CHRU de Tours, Tours, France; 4 Service de néphrologie, Hôpital Necker-enfants malades, Paris, France; 5 Service de néphrologie, Hôpital européen Georges Pompidou, Paris, France; 6 Service de néphrologie, CHU de Clermont-Ferrand, Clermont-Ferrand, France; 7 Service de néphrologie, CHU de Besançon, Besançon, France; 8 Service de néphrologie, CHU d’Angers, Angers, France; 9 Service de néphrologie, CH de Chartres, Chartres, France; 10 Service de néphrologie, CHU de Rennes, Rennes, France; 11 Service de néphrologie, CHU de Brest, Brest, France; 12 Service de néphrologie, CH de La Rochelle, La Rochelle, France; 13 Service de néphrologie et immunologie clinique, Institut de transplantation urologie et néphrologie ITUN, CHU de Nantes, Nantes, France; 14 Service d’anatomie pathologique, Hôpital européen Georges Pompidou, Paris, France; 15 Service d’anatomie pathologique, CHU de Clermont-Ferrand, Clermont-Ferrand, France; 16 Service d’anatomie pathologique, CHU de Besançon, Besançon, France; 17 Service d’anatomie pathologique, CHU d’Angers, Angers, France; 18 Service d’anatomie pathologique, Hôpital Tenon, Paris, France; 19 Service d’anatomie pathologique, CHU de Rennes, Rennes, France; 20 Service d’anatomie pathologique, CHU de Brest, Brest, France; 21 Service d’anatomie pathologique, CHU de Poitiers, Poitiers, France; 22 Service d’anatomie pathologique, CHU de Nantes, Nantes, France; 23 Plateforme IBiSA de Microscopie Electronique, CHRU de Tours, Tours, France; 24 Service d’anatomie pathologique, Hôpital Necker-enfants malades, Paris, France

**Keywords:** Infection, Kidney, Staphylococcus, IgA, Diabetes

## Abstract

**Background:**

Infection-related glomerulonephritis with IgA deposits (IRGN-IgA) is a rare disease but it is increasingly reported in the literature. Data regarding epidemiology and outcome are lacking, especially in Europe. We aimed to assess the clinical, pathologic and outcome data of IRGN-IgA.

**Methods:**

Clinical and outcome data from patients from 11 French centers over the 2007–2017 period were collected retrospectively. We reviewed pathologic patterns and immunofluorescence of renal biopsies and evaluated C4d expression in IRGN-IgA. We analyzed the correlation between histological presentation and outcome.

**Results:**

Twenty-seven patients (23 men, mean age: 62 ± 15 years) were included. Twenty-one (78%) had *Staphylococcus aureus* infection and twelve (44%) were diabetic. At the time of biopsy, 95.2% had haematuria, 48.1% had a serum creatinine level of > 4 mg/dL, and 16% had hypocomplementemia. The most common pathologic presentation included mesangial (88.9%) and endocapillary proliferative glomerulonephritis (88.9%) with interstitial fibrosis and tubular atrophy (IF/TA) (85.1%). Diffuse and global glomerular C4d expression was found in 17.8%, mostly in biopsies with acute or subacute patterns, and was associated with a short delay between infection and renal biopsy compared to segmental and focal staining. After median follow-up of 13.2 months, 23.1% died, 46.2% had persistent renal dysfunction and 15.4% reached end-stage renal disease. Renal outcome was correlated to IF/TA severity.

**Conclusions:**

Infection-related glomerulonephritis with IgA deposits is usually associated with *Staphylococcus* infections and mainly affects adult men. This entity has a poor prognosis which is correlated to interstitial fibrosis and tubular atrophy severity.

## Background

The epidemiology of infection-related glomerulonephritis (IRGN) has changed over the last two decades. Until recently, IRGN mainly comprised poststreptococcal acute post-infectious glomerulonephritis (APIGN) in children [1, 2]. Recent reports indicate that poststreptococcal APIGN still exists in developing countries and in Northern Australia [3, 4]. However, in other countries, IRGN due to *Staphylococcus* is increasingly observed in adults and in the elderly [5–8]. Post-staphylococcal glomerulonephritis (GN) can histologically appear with two patterns: one resembling acute poststreptococcal glomerulonephritis, due to *Staphylococcus aureus* infection and mostly associated with diabetes mellitus, neoplasia or alcoholism; the other with a membranoproliferative glomerulonephritis pattern in *Staphylococcus epidermidis* infection in patients with atrio-ventricular shunts [8, 9]. However, a new presentation was first reported in 1980 by Spector et al. and described in 2003 by Nasr et al. *in* 5 patients with type 2 diabetes, *Staphylococcus aureus* infection, acute renal failure and histologic exudative endocapillary proliferation with predominant mesangial IgA deposits [10]. Since then, American or Asian teams have reported cases and cohorts of infection-related glomerulonephritis with dominant IgA deposits (IRGN-IgA) or codominant with C3 deposits. Nevertheless, the exact epidemiology remains unclear and pathologic findings and outcome of IRGN-IgA have not been described in a large European cohort. The aim of this French nationwide study was to assess the clinical and pathologic aspects and outcome of patients with IRGN-IgA.

## Methods

### Inclusion criteria

Data from 27 patients with IRGN-IgA were collected retrospectively from 11 French hospitals from 2007 to 2017. IRGN-IgA diagnosis was based on the following criteria: 1/proliferative glomerulonephritis (endocapillary and/or mesangial proliferation); 2/IgA deposits in immunofluorescence (IF); 3/clinical diagnosis or laboratory evidence of infection preceding the renal biopsy, with a variable delay between infection and renal biopsy.

The study was approved by the Institutional Ethics Committee in Human Research (No. 2018 008).

### Biopsy specimens

All renal biopsy samples were processed by standard light microscopy and immunofluorescence techniques. They were centrally reviewed by a renal pathologist (E.M.S.) who was blinded to the clinical data. Slides obtained from fixed and paraffin-embedded samples were stained with hematoxylin eosin and saffron, periodic acid-Schiff, trichrome, and Jones or Marinozzi silver. Immunofluorescence was performed in frozen sections using fluorescein isothiocyanate-conjugated antibodies to IgG, IgM, IgA, C3, C1q, kappa, lambda, albumin following the manufacturer’s instructions. Immunohistochemistry was performed in fixed and paraffin-embedded samples using the C4d antibody (clone A24T, prediluted, DB Biotech, Kosice, Slovakia) in a BenchMark XT Platform (Ventana Medical Systems, Oro Valley, Arizona, USA) following the manufacturer’s instructions.

For ultrastructural analysis, biopsies were immersed in a fixative solution of 4% paraformaldehyde and 1% glutaraldehyde in 0.1 M phosphate buffer (pH 7.2) and embedded in Epon resin. Ultrathin sections were cut, stained with 2.5% uranyl acetate, 1% lead citrate, and deposited on gold grids for examination under a transmission electron microscope (TEM) at 100 kV (JEOL 1011, Tokyo, Japan).

### Definition of histologic parameters from renal biopsies

A score was awarded to the following parameters: number of total glomeruli, number of globally sclerotic glomeruli, presence of mesangial hypercellularity (defined as 4 or more cells per mesangial area), segmental (involving < 50% of glomerular capillary tuft) or global (involving ≥50% of glomerular capillary tuft) and focal (involving < 50% of the glomeruli) diffuse (involving ≥50% of the glomeruli) endocapillary proliferation, exudative endocapillary proliferation (defined as endocapillary proliferation of neutrophils), the number of neutrophils per glomerulus (< or ≥ 5), membranoproliferative pattern, crescentic proliferation, fibrinoid necrosis, subepithelial (humps) or intramembranous deposits, interstitial fibrosis with tubular atrophy (IF/TA), interstitial inflammation (both in fibrotic and non-fibrotic cortex), acute tubular injury, presence of red blood cell casts, and arteriosclerosis. Interstitial fibrosis with tubular atrophy, interstitial inflammation and acute tubular injury were defined as absent, mild (< 25% of cortical surface area), moderate (26–50%) or severe (> 50%). Arteriosclerosis was defined as absent, mild (vascular narrowing of up to 25% luminal area by fibrointimal thickening), moderate (26–50%), and severe (> 50%). To evaluate potential prognostic involvement, the intensity of these four histologic features (IF/TA, interstitial inflammation, acute tubular injury, arteriosclerosis) was expressed as 0: absent, 1: mild, 2: moderate and 3: severe.

Renal biopsies were classified into three different histologic patterns, based on Haas et al.’s description: acute (diffuse mesangial and endocapillary proliferation, with 5 or more neutrophils per glomerulus), subacute (diffuse proliferation with mesangial and at least segmental endocapillary hypercellularity but less than 5 neutrophils per glomerulus), and resolving (predominantly mesangial hypercellularity). Crescents and fibrinoid necrosis could be observed in both the acute and subacute patterns but not in the resolving pattern [11, 12].

A score of 0 to 3 was awarded to intensity of IF staining, and the localization (mesangium and/or peripheral capillary loops) of deposits was assessed. C4d immunohistochemistry staining on capillary walls was graded as follows: 0 (absent), 1 (segmental and focal), 2 (global and diffuse) (Additional Figure 1).

### Baseline clinical and biological data

Clinical data included age, sex, comorbid conditions, cause of infection, type of pathogen, clinical presentation. Renal parameters included serum creatinine, proteinuria, serum albumin levels and presence of hematuria. Severe acute renal injury was defined as stage 3 on the Kidney Disease Improving Global Outcomes (KDIGO) classification (acute elevation of serum creatinine level of > 4 mg/dL and/or need for dialysis).

Other parameters were recorded including serum IgA level, C3 and C4 levels, presence of antineutrophil cytoplasmic antibodies (IgG or IgA ANCA) and specific treatments (including antibiotics, steroids and immunosuppressive drugs).

### Follow-up data

Follow-up parameters were recorded from biopsy date to last visit, dialysis or death. Persistent renal dysfunction (PRD) was defined as an estimated glomerular filtration rate (eGFR using CKD-Epi) of < 60 mL/min/1.73m^2^. End-stage renal disease (ESRD) was defined as a duration of dialysis of > 90 days.

### Statistical analysis

Quantitative data are presented as the mean and standard deviation or median and interquartile range. Qualitative data are presented using percentages. Comparisons were made using the Chi square test for qualitative data (or Fisher exact test) and Kruskal-Wallis test for quantitative data. The statistical analysis was performed using GraphPad Prism version 5.0 (GraphPad Software, La Jolla, California, USA). A *P* value < 0.05 was considered as significant.

## Results

### Clinical characteristics

Twenty-seven patients (23 men, 4 women) with a mean age of 62 ± 15 years (range: 5–83) were included (Table 1). Forty-four percent (*n* = 12) of patients had type 2 diabetes, 69.2% (*n* = 18) had hypertension, 52% (*n* = 13) had cardiovascular history (including ischemic heart disease or heart failure). Forty-four percent (*n* = 11) were persistent or former smokers, 37.5% (*n* = 9) had an active chronic alcohol consumption and 9.1% (*n* = 2) had liver cirrhosis. Immunodepression was present in 3 patients (1 patient was treated for lung cancer, 1 patient had myelodysplasia, 1 patient received immunosuppressive medication to treat Crohn’s disease).

**Table 1:** Demographics, predisposing factors to infection and infectious history

Variables	*n* = 27
**Male** (n (%))	23 (85.2)
**Age**, year (mean ± SD)	62 ± 15
**Comorbid conditions**	
Diabetes mellitus (n (%))	12/27 (44.4)
Hypertension (n (%))	18/26 (69.2)
Cardiovascular disease (n (%))	13/25 (52.0)
Active or former smokers (n (%))	11/25 (44.0)
Alcoholism (n (%))	9/24 (37.5)
Liver cirrhosis (n (%))	2/22 (9.1)
Immunosuppressive drug (n (%))	1/27 (3.7)
**Infectious agent**	
*Staphylococcus* (n (%))	21 (77.8)
MRSA (n (%))	4 (14.8)
MSSA (n (%))	16 (59.3)
*Staphylococcus haemolyticus* (n (%))	1 (3.7)
*Morganella morganii* (n (%))	2 (7.4)
*Streptococcus oralis* (n (%))	1 (3.7)
*ESBL-producing Escherichia coli* (n (%))	1 (3.7)
*Enterococcus faecalis* (n (%))	1 (3.7)
*Enterobacteraerogenes* (n (%))	1 (3.7)
*Chlamydia pneumoniae* (n (%))	1 (3.7)
*Corynebacterium amycolatum* (n (%))	1 (3.7)
*Dermabacter hominis* (n (%))	1 (3.7)
More than one pathogen (n (%))	7 (25.9)
Unknown (n (%))	3 (11.1)
**Sites of infection**	
Bone and joint infection (n (%))	12 (44.4)
Skin infection (n (%))	11 (40.7)
Bacteremia (n (%))	11 (40.7)
Other sites	
Prosthesis, plate osteosynthesis or implantable venous access port (n (%))	5 (18.5)
Endocarditis (n (%))	4 (14.8)
Pneumonia (n (%))	4 (14.8)
Urinary tract infection (n (%))	3 (11.1)

*Abbreviations*: *ESBL* extended-spectrum beta-lactamases, *MRSA* methicillin-resistant *Staphylococcus aureus*, *MSSA* methicillin-sensitive *Staphylococcus aureus*, standard deviation

### Infection characteristics

The infectious agent was identified in 88.9% of patients (Table 1).

*Staphylococcus* was the most frequent causative agent (77.8%) (methicillin-sensitive *Staphylococcus aureus* (MSSA) in 59.3% and methicillin-resistant *Staphylococcus aureus* (MRSA) in 14.8%). In 25.9% of the cases, two or more pathogens were identified. A variety of other pathogens were identified including *Streptococcus oralis*, *Chlamydia pneumoniae* or *Escherichia coli*.

Sites of infection were identified in all patients: bone and joint (44.4%) and skin (40.7%) were the most frequent sites. Other infections included prosthesis, plate osteosynthesis, or implantable venous access port infections, endocarditis, pneumonia and urinary tract infection. Bacteremia was present in 40.7% of cases.

### Renal presentation

Renal presentation included nephrotic syndrome for 66.7% of patients, acute nephritic syndrome in 55.6% and rapidly progressing glomerulonephritis in 55.6% of cases (Table 2).

**Table 2:** Renal presentation

**Renal parameters**	
Nephrotic syndrome (n (%))	18/27 (66.7)
Acute nephritic syndrome (n (%))	15/27 (55.6)
Rapidly progressive glomerulonephritis (n (%))	15/27 (55.6)
Hematuria (n (%)) (microscopic/macroscopic)	20/21 (95.2) (11/8)
Serum creatinine, mg/dL (mean ± SD)	4.24 ± 2.93
Creatinine > 4 mg/dL (n (%))	13/27 (48.1)
eGFR, mL/min/1.73m^2^ (mean ± SD (range))	23.7 ± 19.9 (3–82)
Albumin, g/L (mean ± SD (range))	24.7 ± 7.4 (15–42)
Proteinuria, g/day (mean ± SD)	5 ± 3.4 (0.4–16.4)
Baseline serum creatinine, mg/dL (mean ± SD)	1.06 ± 0.3
**Other biological parameters**	
Low C4 levels (n (%))	2/26 (7.7)
Low C3 levels (n (%))	4/25 (16.0)
Both C3 and C4 low levels (n (%))	2/25 (8.0)
High serum IgA levels (n (%))	11/13 (84.6)
ANCA (n (%))	4/15 (26.7)

*Abbreviations*: *ANCA* antineutrophil cytoplasmic antibodies, *eGFR* estimated glomerular filtration rate, *SD* standard deviation

All patients had proteinuria (mean proteinuria: 5 ± 3.4 g/day), 95.2% had hematuria, with macroscopic hematuria in 8 cases. Serum creatinine ranged from 0.99 mg/dL to 13.63 mg/dL (mean: 4.24 ± 2.93) and estimated glomerular filtration rate (eGFR) varied from 3 to 82 mL/min/1.73m^2^. Severe acute renal injury was present in 48.1% of patients and 33% required hemodialysis. Hypocomplementemia was detected in only 16% of patients (both low C3 and C4 levels in 8%). Serum IgA level was increased in 84.6% of the 13 patients tested. Antineutrophil cytoplasmic antibodies (ANCA) were detected in 26.7% of 15 patients.

### Pathology findings

The median delay between clinically apparent onset of infection and biopsy was 42 days (IQR: 26–69). Pathology findings are summarized in Table 3.

**Table 3:** Microscopy findings

Variables	n = 27
**Histologic features**	
**No. of glomeruli** (mean ± SD (range))	15 ± 9 (3–46)
**Globally sclerotic glomeruli** (mean ± SD (range))	2 ± 2 (0–8)
**Mesangial hypercellularity** (n (%))	24 (88.9)
**Endocapillary proliferation** (n (%))	24 (88.9)
Segmental (n (%))/Global (n (%))	9 (33.3)/15 (55.6)
Focal (n (%))/Diffuse (n (%))	10 (37.0)/14 (51.9)
**Exudative endocapillary proliferation** (n (%))	22 (81.4)
< 5 neutrophils per glomerulus (n (%))/≥5 neutrophils per glomerulus (n (%))	15 (55.5)/7 (25.9)
**Membranoproliferative pattern** (n (%))	9 (33.3)
**Crescentic proliferation** (n (%))	10 (37.0)
**Cellular (n(%))/Fibrocellular (n(%))/Fibrous (n(%))**	7 (25.9%)/5 (18.5%)/0
**Fibrinoid necrosis** (n (%))	3 (11.1)
**Deposits** (n (%))	16 (59.2)
Subepithelial humps (n (%))/Intramembranous (n (%))	13 (48.1)/3 (11.1)
**Interstitial fibrosis and tubular atrophy** (n (%))	23 (85.1)
Mild (n (%))/Moderate (n (%))/Severe (n (%))	12 (44.4)/5 (18.5)/6 (22.2)
**Interstitial inflammation** (n (%))	21 (77.8)
Mild (n (%))/Moderate (n (%))/Severe (n (%))	16 (59.3)/5 (18.5)/0
**Acute tubular injury** (n (%))	23 (85.1)
Mild (n (%))/Moderate (n (%))/Severe (n (%))	8 (29.6)/8 (29.6)/7 (25.9)
**Red blood cells casts** (n (%))	18 (66.7)
**Arteriosclerosis** (n (%))	24 (88.9)
Mild (n (%))/Moderate (n (%))/Severe (n (%))	4 (14.8)/16 (59.3)/4 (14.8)
**Histologic pattern**	
**Acute** (n (%))	7 (25.9)
**Subacute** (n (%))	17 (63.0)
**Resolving** (n (%))	3 (11.1)

*Abbreviations*: *SD* standard deviation

**Fig. 1:**
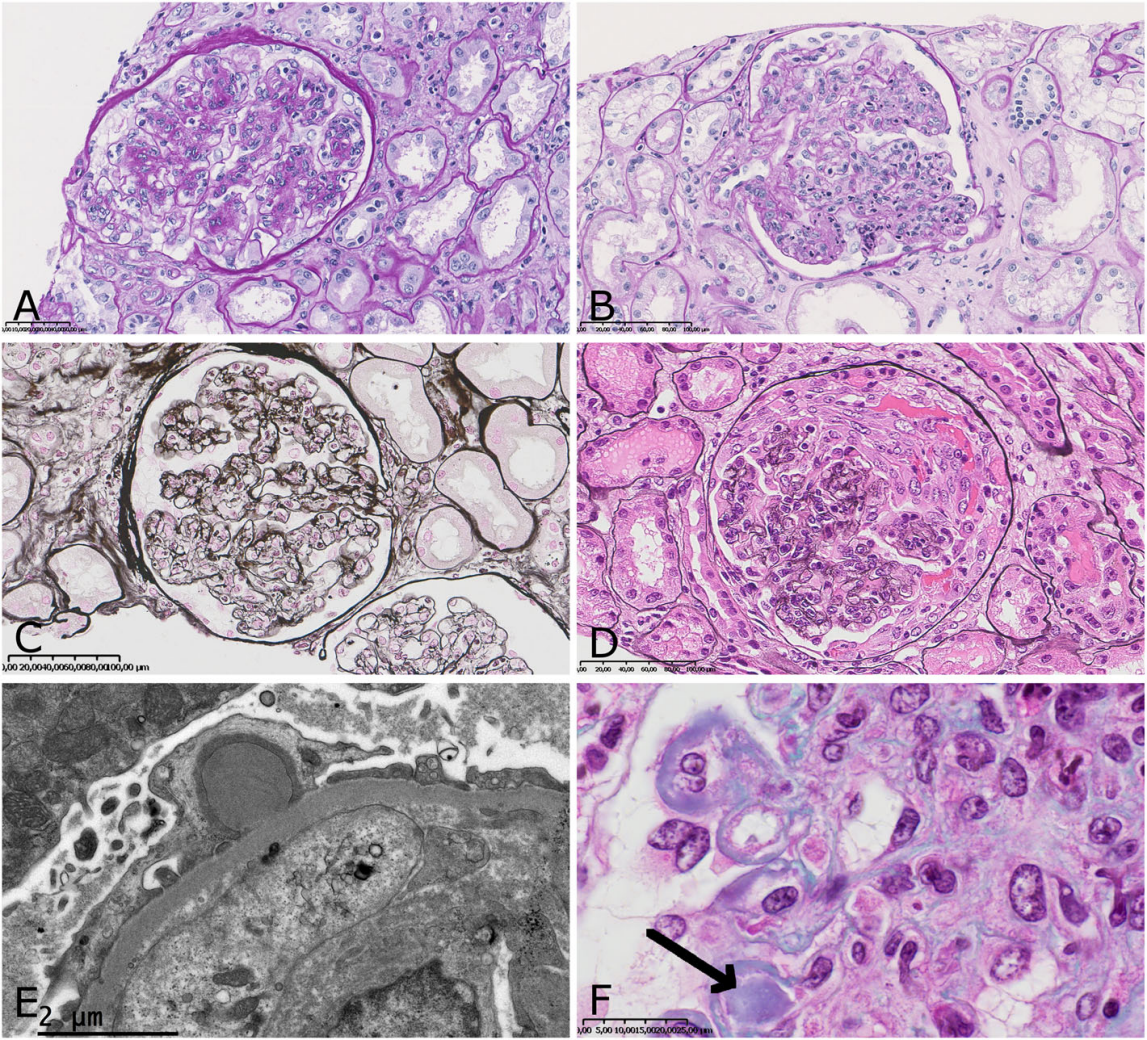
Microscopy. **a**: mesangial with endocapillary proliferation was the most frequent pattern (Jones silver stain × 300); **b**: exudative endocapillary proliferation (PAS stain × 200); **c**: membranoproliferative glomerulonephritis (Jones silver stain × 200); **d**: crescentic proliferation with fibrinoid necrosis (Jones silver stain × 300); **e**: subepithelial humps deposit (electron microscopy × 20.000); **f**: subendothelial deposits with hyaline thrombus resembling cryoglobulin (arrow) (Masson’s trichrome stain × 1000)

Endocapillary proliferation associated with mesangial proliferation was the most frequent pattern (81.5%) (Fig. 1a). Mesangial proliferation was pure in 7% of cases. Endocapillary proliferation most frequently involved neutrophils (81.4%) (Fig. 1b). In one patient we observed only globally sclerotic glomeruli without proliferation. Membranoproliferative pattern and crescentic proliferation were also observed (33.3 and 37% respectively) (Fig. 1c and d). All biopsies with crescent formation had endocapillary proliferation and almost all (9 out of 10) had mesangial proliferation. In almost all biopsies we observed de novo proliferation, except in one case (4%) in which proliferation was superimposed on diabetic nephropathy. We identified subepithelial humps deposits in 48.1% of biopsies and prominent deposits in the glomerular capillary wall of 11.1% of biopsies, with hyaline thrombi resembling cryoglobulin in one (Fig. 1e and f). Interstitial fibrosis and tubular atrophy (IF/TA) were observed in 85.1% of cases (44.4% mild, 18.5% moderate, 22.2% severe). Classification according to pattern presentation revealed 25.9% acute, 63% subacute and 11.1% resolving GN (Table 3).

### Immunofluorescence and immunohistochemistry

Immunofluorescence features are summarized in Table 4.

**Table 4:** Immunofluorescence and immunohistochemistry findings

Immunofluorescence	
IgA	27/27 (100)
+/ ++ / +++ (n (%))	8 (29.6)/9 (33.3)/10 (37.1)
Mesangial/Capillary loop/Both (n (%))	9 (34.6)/5 (19.2)/12 (46.2)
C3	27/27 (100)
+/++/+++ (n, %)	5 (19.2)/6 (23.1)/15 (57.7)
Mesangial/Capillary loop/Both (n (%))	9 (36.0)/2 (8.0)/14 (56.0)
IgA and C3 codominant (n (%))	15 (55.5)
IgA dominant (n (%))	3 (11.1)
IgG staining (n (%))	4/27 (14.8)
IgM (n (%))	6/27 (22.2)
C1q (n (%))	0
Kappa (n (%))	9/24 (37.5)
Lambda (n (%))	14/24 (58.3)
**C4d Immunohistochemistry**	23/27 (85.2)
0/+/++ (n (%))	8 (34.8)/11 (47.8)/4 (17.4)

IgA granular deposits were observed in all biopsies with various locations: mesangium (34.6%), both mesangium and peripheral capillary loops (46.2%) or capillary loops only (19.2%). A “starry sky” pattern was noticed in 4 cases (15%). C3 deposits were observed in 100% of biopsies. Dominant IgA deposits were observed in 3 cases (11.1%) or most frequently codominant with C3 (55.5%). C1q deposits were not identified.

Immunohistochemistry with C4d antibody was performed in 23 biopsies of IRGN-IgA. Most of the biopsies had focal glomerular C4d 1+ staining (47.8%) or no glomerular staining (C4d0, 34.8%). C4d 2+ glomerular staining was only seen in 4 biopsies (17.4%). The 4 biopsies exhibited an acute (*n* = 2) or subacute (n = 2) pattern.

### Renal characteristics according to histologic pattern

**Fig. 2:**
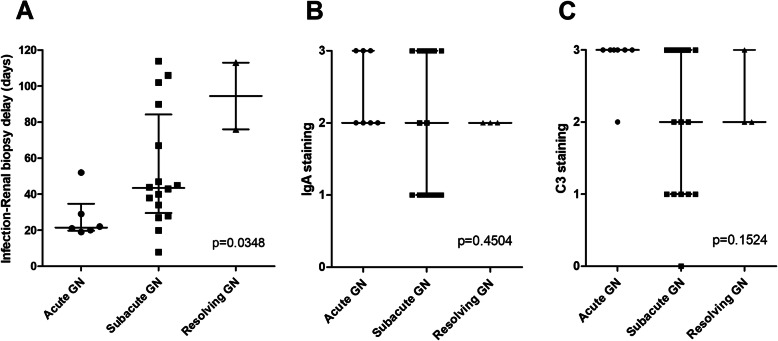
Infection-to-biopsy delay and immunofluorescence according to histologic pattern. **a**: Delay in days between documentation of infection and renal biopsy according to histologic pattern; **b**: IgA intensity staining according to histologic pattern; **c**: C3 intensity staining according to histologic pattern. Values are expressed as median and interquartile range. Abbreviations: GN: glomerulonephritis

The delay between infection and renal biopsy, available for 88.9% of patients, was significantly increased according to the glomerulonephritis pattern from acute GN (median: 21.5 days, IQR: 20.3–27.3) to subacute (median: 43.5 days, IQR: 32.5–72.8) and resolving GN (median: 94.5 days, IQR: 85.3–103.8) (*P* value = 0.03) (Fig. 2a). We observed that the delay between infection and renal biopsy was shorter in C4d 2+ stained biopsies (median: 24.5 days) compared to C4d 0 and C4d 1+ stained biopsies (median: 43 and 45 days respectively, *P* value = 0.05). We did not observe any differences in IgA staining according to histologic pattern (median: 2+ for all groups) but C3 staining was higher in patients with acute GN (median: 3+) than in those with subacute and resolving GN (median: 2+) (Fig. 2b and c).

Comparing the three histologic patterns (acute, subacute and resolving), we saw that the percentage of skin infections tended to be more frequent in the acute than in the subacute and resolving groups (respectively 46.2% vs 14.8 and 20%, *P* value = 0.09) whereas bone and joint infections tended to be less frequent in the acute group (7.7% vs 33.3 and 40% respectively, *P* value = 0.1) (data not shown).

### Therapeutic management and outcome

All patients received antibiotics (Additional Table 1). The antibiotics the most commonly used were penicillin (77.8%) and rifampicin (40.7%), and 88.9% of patients received two or more antibiotics. Other antibiotics included cephalosporin, aminoside, macrolide, quinolone, glycopeptides and carbapenem. In addition to antibiotics, corticosteroids were used in 37% of patients.

Clinical follow-up was available in 26 out of 27 patients (96.3%) with median follow-up of 13.2 months (IQR: 4.0–22.2) (Additional Table 2).

At last follow-up, 23.1% of patients had died, 46.2% had persistent renal dysfunction, and 15.4% had ESRD. One patient died due to progression of pulmonary carcinoma, one died of aspiration pneumonia, another of septic shock. For the 3 other patients, the cause of death was not available. For one dead patient information about eGFR was not available, and 25 patients (92.5%) could be classified according to their eGFR at follow-up (Table 5): 28% had eGFR> 60 mL/min/1.73m^2^, 56% had persistent renal disease (PRD) and 16% had end-stage renal disease (ESRD).

**Table 5:** Outcome and prognostic factors (25 patients)

	eGFR > 60 mL/min/1.73m^2 a^	PRD ^b^	ESRD ^c^	*P* values
**No. of patients**	7	14	4	
**% of patients**	28	56	16	
**Age, year**	61	64	68	0.8
**Median follow-up, months**	8.3	16.5	21.6	0.3
Mean eGFR, ml/min/1.73m^2^				
At biopsy	28.9	20.9	17.5	0.6
Follow-up	84.6	37.5	–	**< 0.001**
Mean proteinuria at biopsy, g/day	3.5	5.5	6.5	0.2
Corticosteroids, % of patients	0	64	25	**0.01**
Median infection-renal injury delay, days (IQR)	13 (8.5–36)	23 (17.8–69)	13 (10–47.3)	0.3
Histologic pattern (acute/subacute/resolving, %)	29/57/14	29/64/7	50/25/25	0.1
Global glomerulosclerosis, % of glomeruli	13	18	44	0.2
Crescentic proliferation, % of glomeruli	4	6	5	0.9
Interstitial inflammation score, mean	0.9	0.9	1.5	0.2
Acute tubular injury score, mean	1.6	1.8	1.75	0.8
IF/TA score, mean	0.9	1.6	2.5	**0.02**
Arteriosclerosis, mean	2	1.8	2	0.6

*Abbreviations*: *eGFR* estimated glomerular filtration rate, *ESRD* end-stage renal disease, *IF/TA* interstitial fibrosis with tubular atrophy, *IQR* interquartile ranges, *PRD* persistent renal disease (eGFR< 60 ml/min/1.73m^2^)

^a^One patient with eGFR> 60 died; ^b^One patient with PRD died; ^c^Two patients with ESRD died

In univariate analysis, there was no significant correlation between renal outcome and age, eGFR at biopsy, proteinuria at biopsy, or histologic pattern (acute/subacute/resolving pattern). The only association between histologic findings and renal outcome was related to IF/TA: the IF/TA score was significantly higher in the PRD group (IF/TA score = 1.6) and ESRD (IF/TA score = 2.5) groups compared to the eGFR> 60 mL/min (IF/TA score = 0.9) group (*P* value = 0.02).

## Discussion

Infection-related glomerulonephritis with IgA deposits, rarely reported in Europe [13, 14], affects mostly patients who present with staphylococcal infection, hematuria, proteinuria and acute kidney injury with a proliferative glomerulonephritis. This presentation is comparable to that observed in American and Asian populations with some particularities in European patients. Moreover, the wide spectrum of both clinical presentation and histologic pattern can make the diagnosis challenging [15].

In our cohort, most patients affected were males over 60. This finding is comparable to previous results reporting that 75 to 86% of the patients are male with a mean age of 55 to 65 years [9, 10, 12, 16–20]. It must be noted that the youngest of our patients is a 5-year-old boy, indicating that IRGN-IgA, although rarely reported, can be observed in pediatric patients. In this case and in other reported pediatric cases, children with *Staphylococcus*-related GN had the same presentation as adults, namely proteinuria and renal function impairment [21].

Poststaphylococcal GN can occur in various immunocompromised backgrounds and a poor prognosis is mainly related to age and comorbidities [5, 6]. The initial description by Nasr et al. reported diabetic nephropathy in all biopsies. Nevertheless, the association between diabetes and IRGN-IgA is inconstant, reported in 8 to 55% of patients in previous studies and in 44% of patients in our study [10, 12, 16–18, 22–24]. As seen since the first report, *Staphylococcus* represents the most frequent germ (78% in our study, 60 to 100% in other studies) [10, 12, 17, 18, 24]. A higher frequency of MRSA was observed in Asian and American studies (50 to 60%) compared to our cohort (15%). This observation is consistent with the low incidence of MRSA observed in France [25].

Regarding the histological features of IRGN-IgA, we noticed some differences compared to Asian and American studies. Most of them reported a similar proportion of mesangial proliferation, crescentic proliferation and fibrinoid necrosis. In our study, endocapillary proliferation (89% vs 23 to 63% in previous series) involved neutrophils in most cases (81% vs 15 to 63% in previous series) and was more frequent than pure mesangial proliferation [9, 10, 12, 17, 18, 20]. We also noticed differences when comparing histological patterns as classified in acute, subacute and resolving by Haas et al. in 2008 [12]. The authors reported more resolving (62%) and less acute (15%) or subacute (23%) patterns compared to our cohort (26, 63 and 11% respectively). It is possible that the time between infection and renal biopsy was shorter in these patients than in patients from other cohorts. The relationship between the infection-to-biopsy delay and histological pattern supports the concept that these patterns represent different evolving aspects of the same disease.

C4d staining is not routinely performed on native kidney biopsy, however it is increasingly studied in various types of glomerulonephritis. We assumed that C4d staining could provide additional information about complement activation in IRGN-IgA. C4d deposits were observed in 65% of our biopsies. Diffuse and global (C4d 2+) capillary wall staining was observed in biopsies with proliferative pattern (acute and subacute), and with a shorter delay between infection and biopsy assessment. These observations are in favor of the activation of the complement pathway, at least during the active phase of infection. According to Sethi et al., C4d deposits in infection-related GN could be related to activation of the classical pathway or lectin pathway of complement [26]. However, in a significant number of cases (34% in our study, 46% in their study), no C4d deposits were observed. Two mechanisms can be proposed: first, complement alternative pathway could be abnormally activated in some patients; second, it is possible that the infection was no longer active in patients in the time elapsing between the onset of infection and the renal biopsy. The latter hypothesis is supported by the longer time lapse observed in patients with segmental and focal or no deposits compared to patients with diffuse and global C4d staining.

Regarding deposits, in addition to subepithelial “humps” deposits which are commonly described in IRGN-IgA, we also observed large subendothelial deposits with hyaline thrombi in 11% of the biopsies. These deposits are rarely encountered but were previously reported by Satoskar et al. in one biopsy, by Worawichawong et al. in 2 biopsies, and by Khalighi et al. in 5 biopsies [18, 24, 27]. This cryoglobulin-like presentation most frequently occurred in patients with *Staphylococcus aureus* infection. This leads Khalighi et al. to suggest a potential role of staphylococcal toxin as a superantigen responsible for activation of B cells and production of antibodies. Khalighi et al. reported 20% deaths and 80% end-stage renal disease in patients with cryoglobulin-like features (vs 63% of ESRD in patients without cryoglobulinemic presentation). In our cohort, 66% (2 out of 3) of patients with cryoglobulinemic features died (vs. 13% in non-cryoglobulinemic), and none of them had an eGFR> 60 mL/min/1.73m^2^ (vs. 26% in non-cryoglobulinemic). This presentation probably corresponds to more severe presentation of IRGN-IgA.

IRGN-IgA is a renal disease with poor prognosis. According to the literature data, the risk of end-stage renal disease varies from 20 to 80% of patients, and risk of death reaches 30% [5, 12, 18, 20]. In our study, 33% of patients required hemodialysis during the acute phase of GN, 15% the patients progressed to ESRD, and 23% died. Some authors observed that patients with renal recovery had less frequent acute tubular injury, interstitial inflammation or IF/TA [5, 18]. Our results confirmed the association between the severity of IF/TA and renal prognosis in French patients.

Whether treatment with corticosteroids modified the outcome is unsure. Ten patients (37%) in our study received corticosteroids in addition to antibiotics, and were patients with the more severe renal presentation. No significant improvement in renal outcome was observed. The use of corticosteroids remains controversial. For some authors [28], steroids may have a place in the treatment of patients who fail to respond to antibiotic therapy or patients with crescentic proliferation, whereas for other authors [29] it can be deleterious in this form of GN in which infection is often ongoing.

IgAN and IRGN-IgA have similarities which may lead to misdiagnosis, particularly when infections are undiagnosed for a long time. Satoskar et al. summarized both clinical and histologic features that could be helpful in distinguishing these two diagnoses [18, 19]. In our cohort, arguments in favor of IRGN-IgA are: older age (89% were over 50 vs < 30 in most patients with IgAN); nephrotic range proteinuria (70% in our cohort but rare in patients with IgAN); low C3 (16% in our cohort and usually normal in IgAN); severe acute renal failure (all cases except for one in our cohort but uncommon in IgAN). Of note, C4 staining could be an additional distinctive point, it remains classically negative in IgAN because of the activation of the alternative pathway of complement [26]. We performed C4d staining in a small number of supplemental biopsies including 7 classical postinfectious GN and 9 IgAN (data not shown). Interestingly, none of these 16 biopsies presented diffuse capillary wall C4d staining, leading us to speculate that the presence of capillary wall C4d staining could help to distinguish both entities. However, C4d staining was previously observed in some cases of IgAN, corresponding to the activation of lectin pathway of complement, and predictive of a poorer prognosis [30]. A recent study reported 26.4% of capillary wall C4d staining in IgAN, correlated to endocapillary proliferation, rendering C4d inaccurate for differential diagnosis with IRGN-IgA [31]. Some authors hypothesized that IgAN can develop secondary to *Staphylococcus aureus* infection [32]. However, this hypothesis does not explain the pathophysiology of IRGN-IgA due to other pathogens (25% of our cohort).

IgA deposits due to liver disease represent another differential diagnosis for IRGN-IgA. Most of the previous series of IRGN-IgA included patients with chronic hepatic disease. The largest series reported by Satoskar et al., included 28% of patients with hepatitis C, and at least two of them had liver cirrhosis [19]. According to Hemminger et al., the autopsy series report incidental IgA in about 65% of patients with cirrhosis, but this finding is not associated with renal dysfunction [33]. In our cohort, the 2 patients with hepatic cirrhosis had acute renal failure. They hypothesized that glomerular IgA deposits may be due to both excessive immune complex deposition secondary to bacterial infection and poor clearance secondary to liver dysfunction. The mechanisms, not yet understood, require further pathophysiologic explorations.

## Conclusions

Immune deposit-associated glomerulonephritis is a rare and usually severe infection-associated GN that mostly occurs in patients over 60 with nephrotic range proteinuria, hematuria and/or rapidly progressive glomerulonephritis. Various patterns can be observed but acute endocapillary proliferation seems more frequent in our French cohort than in other American and Asian cohorts and is associated with global and diffuse C4d staining. This entity may be difficult to distinguish from IgA nephropathy particularly in patients with a histologic resolving pattern. The global and renal outcome remains poor.

## Supplementary information

**Additional file 1: Figure 1**: C4d immunohistochemistry.

**Additional file 2: Table 1**: Treatments.

**Additional file 3: Table 2:** Follow-up and renal outcome.

## Data Availability

The datasets used and/or analyzed during the current study are available from the corresponding author on reasonable request.

## Abbreviations

ANCA: Antineutrophil cytoplasmic antibodies
APIGN: Acute post-infectious glomerulonephritis
eGFR: Estimated glomerular filtration rate
ESRD: End-stage renal disease
GN: Glomerulonephritis
IF: Immunofluorescence
IF/TA: Interstitial fibrosis with tubular atrophy
IQR: Interquartile range
IRGN: Infection-related glomerulonephritis
IRGN-IgA: Infection-related glomerulonephritis with IgA deposits
KDIGO: Kidney Disease Improving Global Outcomes
MRSA: Methicillin-resistant *Staphylococcus aureus*
MSSA: Methicillin-sensitive *Staphylococcus aureus*
