# Supplementary figures and images for: Clinicopathologic features of infection-related glomerulonephritis with IgA deposits: a French Nationwide study
Source: Diagn Pathol. 2020 May 27;15:62. doi: 10.1186/s13000-020-00980-6 (PMC7254713; doi:10.1186/s13000-020-00980-6)

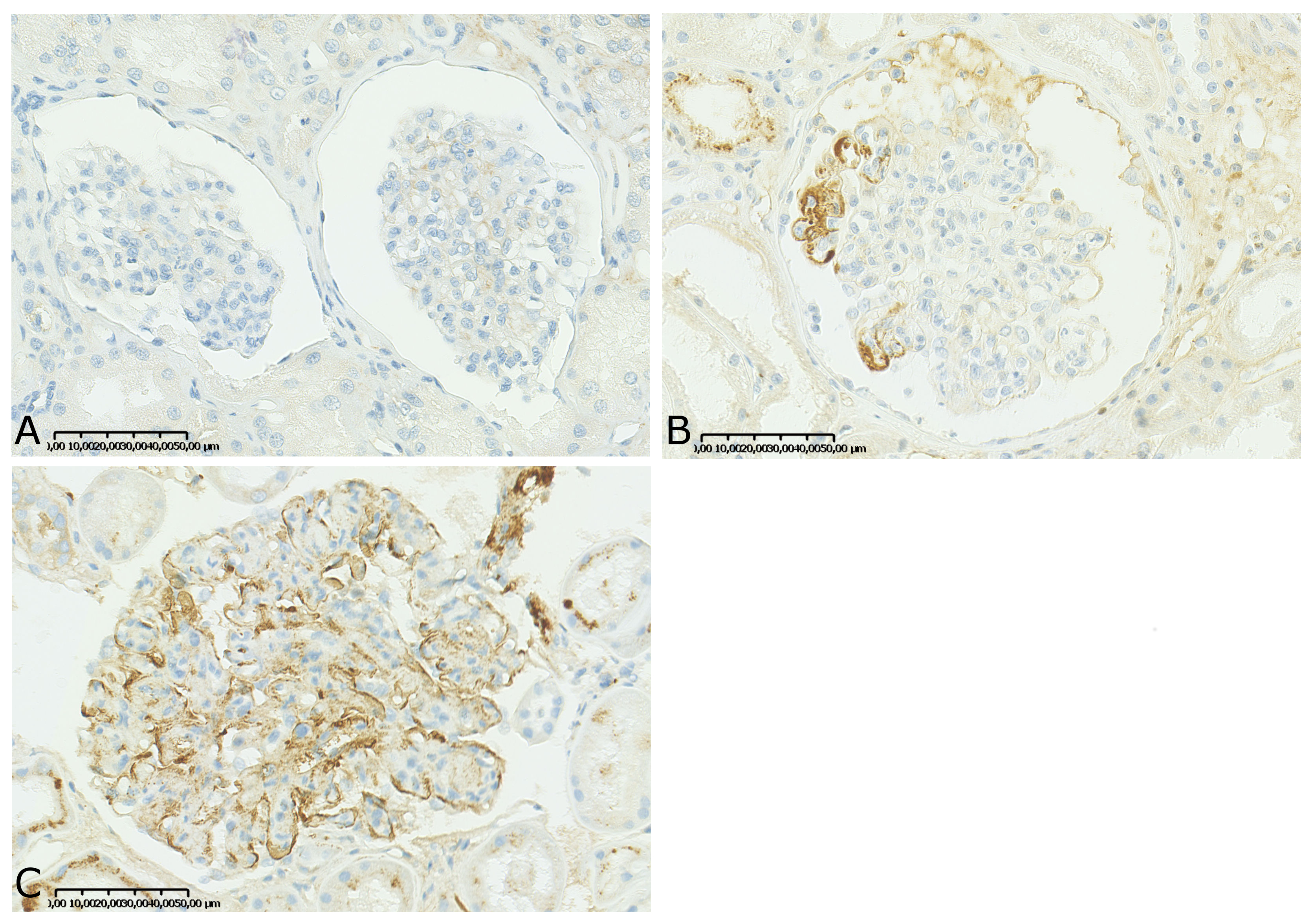

Supplement: Supplementary file 1 — Additional file 1: Figure 1: C4d immunohistochemistry. [file 13000_2020_980_MOESM1_ESM.jpg]
