# Supplementary material for: Clinicopathologic features of infection-related glomerulonephritis with IgA deposits: a French Nationwide study
Source: Diagn Pathol. 2020 May 27;15:62. doi: 10.1186/s13000-020-00980-6 (PMC7254713; doi:10.1186/s13000-020-00980-6)
Supplement: Supplementary file 2 — Additional file 2: Table 1: Treatments. [file 13000_2020_980_MOESM2_ESM.docx]

**ADDITIONAL DATA**

**Table 1**: Treatments.

| **Antibiotics** | 27/27 (100) |  |
| --- | --- | --- |
| Penicillin (n (%)) | 21/27 (77.8) |  |
| Rifampicin (n (%)) | 11/27 (40.7) |  |
| Cephalosporin (n (%)) | 9/27 (33.3) |  |
| Aminoside (n (%)) | 9/27 (33.3) |  |
| Macrolide (n (%)) | 9/27 (33.3) |  |
| Quinolone (n (%)) | 8/27 (29.6) |  |
| Glycopeptides (n (%)) | 5/27 (18.5) |  |
| Penem (n (%)) | 3/27 (11.1) |  |
| ≥ 2 antibiotics (n (%)) | 24/27 (88.9) |  |
| **Corticosteroids** (n (%)) (oral/pulse) | 10 (37.0) (10/1) |  |
| **Acute dialysis** (n (%)) | 9 (33.3) |  |
