# Supplementary material for: Clinicopathologic features of infection-related glomerulonephritis with IgA deposits: a French Nationwide study
Source: Diagn Pathol. 2020 May 27;15:62. doi: 10.1186/s13000-020-00980-6 (PMC7254713; doi:10.1186/s13000-020-00980-6)
Supplement: Supplementary file 3 — Additional file 3: Table 2: Follow-up and renal outcome. [file 13000_2020_980_MOESM3_ESM.docx]

**ADDITIONAL DATA**

**Table 2:** Follow-up and renal outcome.

Abbreviations: eGFR: estimated glomerular filtration rate, IQR: interquartile ranges, SD: standard deviation

| **After 3 months** |  |
| --- | --- |
| Serum creatinine, µmol/L (mean ± SD) | 242 ± 215 (44-700) |
| Proteinuria, g/day (mean ± SD) | 2.2 ± 2.8 (0.4-11) |
| eGFR, mL/min/1.73m² (mean ± SD (range)) | 42.3 ± 27.6 (5-108) |
| **After 12 months** |  |
| Serum creatinine, µmol/L (mean ± SD) | 163 ± 116 (62-500) |
| Proteinuria, g/day (mean ± SD) | 1.7 ± 2 (0.1-5.4) |
| eGFR, mL/min/1.73m² (mean ± SD (range)) | 51.7 ± 27 (6-94) |
| **Last visit (n (%))** | **26/27 (96.3)** |
| Follow-up time, months (median (IQR)) | 13.2 (4.0-22.2) |
| Persistent renal dysfunction (n (%)) | 12 (46.2) |
| End-stage renal disease (n (%)) | 4 (15.4) |
| Death (n (%)) | 6 (23.1) |
